# Utilization of Pulsed Current‐Electro Fenton Technology for the Treatment of Wastewater from Industrial Processes

**DOI:** 10.1002/open.202400505

**Published:** 2025-02-21

**Authors:** Perumal Asaithambi, Abdelrahman O. Ezzat, Firomsa Bidira, Mamuye Busier Yesuf, Omar H. Abd‐Elkader, Arun Thirumurugan, N. M. Hariharan, Hamad A. Al‐Lohedan, Abudukeremu Kadier

**Affiliations:** ^1^ Faculty of Civil and Environmental Engineering Jimma Institute of Technology Jimma University Jimma Ethiopia Po Box – 378; ^2^ Department of Chemistry College of Sciences King Saud University Po Box 2455 Riyadh 11451 Saudi Arabia; ^3^ Department of Physics and Astronomy College of Sciences King Saud University Riyadh 11451 Saudi Arabia; ^4^ Sede Vallenar, Universidad de Atacama Costanera #105 Vallenar 1612178 Chile; ^5^ Department of Biotechnology Vel Tech Rangarajan Dr. Sagunthala R & D Institute of Science and Technology, Avadi Chennai 600062, Tamil Nadu India; ^6^ Laboratory of Environmental Science and Technology, The Xinjiang Technical Institute of Physics and Chemistry Key Laboratory of Functional Materials and Devices for Special Environments Chinese Academy of Sciences Urumqi 830011 China; ^7^ Center of Materials Science and Optoelectronics Engineering University of Chinese Academy of Sciences Beijing 100049 China

**Keywords:** H_2_O_2_, Electro–Fenton, Pulsed, Wastewater, Elimination of pollutant, Consumption of power

## Abstract

In recent years, there has been a rise in the use of electrochemical and advanced oxidation methods to treat the industrial wastewater. The efficiency of several approaches for treating industrial wastewater, including hydrogen peroxide (H_2_O_2_), electro–Fenton (EF) and pulsed–electro–Fenton (PEF) processes were all investigated. In evaluation to the H_2_O_2_, EF, and PEF technologies, the results showed that the PEF process produced 100 % total color and 98 % chemcial oxygen demand (COD) removal efficiency with a low consumption of power of 3.4 kWhrm^−3^. The experimental parameters comprised the following: COD – 2500 mg L^−1^, pH – 3, H_2_O_2_ – 300 mg L^−1^, distance between electrode – 0.75 cm, current – 0.40 A, cycle of pulse duty – 0.75, combination of electrode – Fe/Fe, stirring speed – 500 rpm and treatment duration (TD) – 125 min. It was demonstrated that increasing the TD, current, and H_2_O_2_ while lowering the COD content improved the COD elimination efficiency while employing a iron (Fe/Fe) electrode combination with wastewater pH of 3. The efficiency of the EF process has been reduced in comparison to the PEF process because of the development of an impermeable oxide layer on the cathode and the oxidation–induced corrosion on the anode. Consequently, experimental results have indicated that the PEF could be a more promising technology than the EF method for eliminating pollutants from wastewater with reduced power consumption and process efficiency.

## Graphical abstract

**Pulsed‐Electro‐Fenton method**: Experiments were conducted on the direct and pulsed current‐electro‐Fenton processes. EAOP methods were compared for elimination color and COD from industrial wastewater. EF technique effectively removes color and COD while consuming minimal electricity. Operating parameters were evaluated to improve process efficiency.

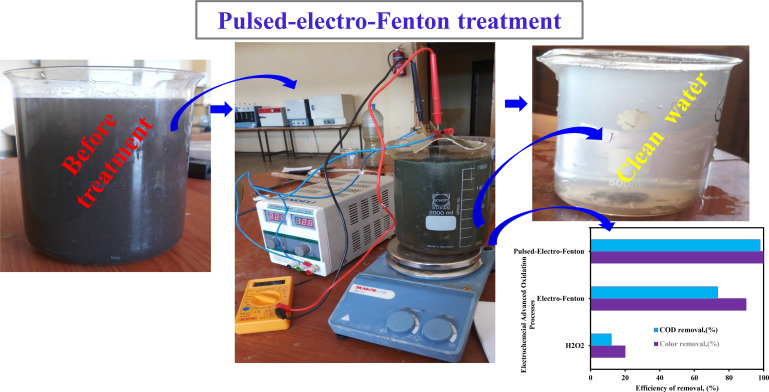

## 1 Introduction

Significant attention has been devoted to the electrochemical (EC) method for the treatment of wastewater from various sectors, including composite industrial,^[1]^ molasses‐based alcohol distillery,^[2]^ toxic,^[3]^ hospital,^[4]^ sanitizer industry,^[5]^ organic pollutants,^[6]^ water treatments,^[7]^ baker's yeast,^[8]^ degradation of organic pollutants,^[9]^ colored textile,^[10]^ pulp and paper,[^11^, ^12^] tannery,^[13]^ pesticide,^[14]^ oily,[^15^, ^16^] metal‐plating,^[17]^ dairy,^[18]^ shipyard,^[19]^ reactive blue 19 synthetic,^[20]^ slaughterhouse,^[21]^ domestic,^[22]^ landfill leachate,^[23]^ municipal,^[24]^ dye bath,^[25]^ marble processing,^[26]^ and etc. Compared to other conventional methods, the EC process provides several advantages, including easy experimental setup and operation, shorter treatment times, no chemical additions, faster floc sedimentation and less sludge development, and high pollution removal efficiency with less electricity consumption.[^27^, ^28^] Anodic oxidation is the source of the electro‐Fenton (EF) process, which is characterized by the release of metal ions (Fe^2+^ or Fe^3+^, Al^3+^). This is accomplished by the utilization of sacrificial soluble iron (Fe) and/or aluminum (Al) as anodes and/or cathodes, respectively. Afterwards, these ions combine with the hydroxyl ions that are released from the cathode, leading to the formation of hydroxides of Fe or Al and encouraging the particles to aggregate into flocs.^[29]^

Table 1 shows how well the EF procedure reduces pollutants in different types of wastewater. According to the data shown in Table 1, the EF technique is more effective at eliminating contaminants from all kinds of wastewater. Nevertheless, there is a scarcity of research on the pulsed–Electro–Fenton (PEF) process ability to reduce pollutants and calculate the consumption of power of industrial effluent from distilleries. It is common practice in the EF process to make use of direct–current (DC) for the purpose of treating a wide variety of wastewater–related substances.[^30^, ^31^] The primary limitations of the direct–current–electro–Fenton (DC–EF) technology are the inescapable formation of an impermeable oxide layer on the cathode and the presence of corrosion on the anode as a result of oxidation.[^1^, ^12^, ^32^, ^33^] These factors hinder the efficient flow of electrical current between the anode and cathode. As a result, the DC–EF method was abandoned because its results showed lower pollutant removal effectiveness and higher energy and operational costs.^[34]^ The alternating–current–electro–Fenton (AC–EF or PEF) approach or frequent anode or cathode replacement in DC mode operation are two ways to lessen the drawback of the DC–EF process with cathode passivation.^[35]^

**Table 1:** Comparison of the pollutant reduction efficiency by electro‐Fenton process with types of wastewater.

Wastewater Types	Optimum conditions	Removal Efficiency	Literature
Ponceau 4R	pH=3, voltage=20 V, H_2_O_2_=0.033 % v/v, Na_2_SO_4_=0.1 M and the C_P4R_=50 mg L^−1^, treatment duration=10 min	P4R‐100 %, energy consumed – 19.82 KWh m^−3^	[71]
Refinery	pH=3, temperature=60 °C, and electrolysis time=30 min	Organic removal – 93.45 %	[72]
Reactive yellow 145	Voltage=9.4 V, pH=3.7, electrode spacing=2.5 cm, H_2_O_2_=1 mL and treatment time=40 min	Dye – 93 %, energy consumption – 3.4 Wh L^−1^	[71]
Dairy	pH=5.95, reaction time=60 min, H_2_O_2_=1.5 ml and molar H_2_O_2_/Fe^2+^=1.8	COD – 95.8 %, turbidity – 97.2 %.	[73]
Reactive Orange 16 Dye	pH=3.5, initial RO16 135=mg L^−1^, electrolysis time=42.5 min, and current density=17.5 mAm^−2^	Decolorization – 72 % and COD – 61 %	[74]
Spent caustic	H_2_O_2_/COD=0.73, pH=5.46, current density=18.83 mA cm^−2^ and treatment time=69.88 min	COD – 93.0 %	[75]
Petroleum refinery	Current density=10.4545 mA cm^−2^, Fe^2+^=0.50 mM, reaction time=57.57 min	COD – 91.64 %, SEC – 5.96 KW h kgCOD^−1^	[76]
Hospital	current density=19.293 mA cm^−2^, H_2_O_2_/COD ratio=1.4, time duration=46.67 min	COD – 96.27 %, energy – 7.325 kWh kg COD^−1^	[77]
Textile industry	pH=3, current intensity=1.65 A, Fe^2+^=2 mM and flow rate=25 mL min^−1^	Colour – 89 %, COD – 93 %, TOC – 58 %	[78]

Previous studies have demonstrated the advantages of using alternating–current–EC (AC–EC) techniques to reduce wastewater contaminants such as distillery industrial effluent,^[36]^ cadmium,^[37]^ brewery,^[34]^ synthetic and real smelting,^[38]^ lead and zinc,^[39]^ copper,^[40]^ aqueous solution,^[41]^ and etc. Prior studies have demonstrated the advantages of employing an AC–EC approach to lower contaminants in synthetic wastewater. Concurrently, there has been a scarcity of research conducted on actual industrial wastewater.^[42]^ In addition to its practical and economic significance, electrical energy usage is an essential component of AC–EC procedures since it helps in the removal of pollutants from industrial effluent.

### 1.1 Mechanisms of EF/PEF Processes

The commonly accepted technique for removing impurities by EF using Fe electrodes is as follows.[^43^, ^44^, ^45^]

Anodic reaction:

(1) $${Fe}_{\left(s\right)}\to {Fe}_{\left(aq\right)}^{2+}+2e^{-}\;\;\;E^{0}=-0.44V\;vs\;SHE$$

Cathodic reaction:

(2) $${2H}_{2}O_{\left(l\right)}+2e^{-}\to \;H_{2\left(g\right)}+2OH_{\left(aq\right)}^{-}\;\;\;E^{0}=0.83V\;vs\;SHE$$

Overall reaction:

(3) $${Fe}_{\left(s\right)}+2H_{2}O_{\left(l\right)}\to Fe({OH)}_{2\left(s\right)}+H_{2\left(g\right)}$$

The dissolved *Fe* ^*2+*^ oxidizes and becomes insoluble *Fe(OH)_3_* when oxygen is present.

(4) $$4{Fe}_{\left(aq\right)}^{2+}+10H_{2}O_{\left(l\right)}+O_{2\left(g\right)}\to 4\;Fe{\left(OH\right)}_{3\left(s\right)}+8H_{\left(aq\right)}^{+}$$

By complexation or electrostatic attraction followed by coagulation, the *Fe(OH)*_*n(s)*_ that remain in the aqueous stream as a gelatinous suspension can remove the contaminants from the wastewater.

An EF system uses a sacrificial *Fe* anode as the source of *Fe* ^*2+*^ and introduces *H_2_O_2_* into the system.[^43^, ^44^]

(5) $${Fe}^{2+}+H_{2}O_{2}\to {Fe}^{3+}+HO^{•}+OH^{-}$$

This reaction propagates primarily through the regeneration of ferrous ions, which is accomplished by reducing the resulting ferric species with *H_2_O_2_*.

(6) $${Fe}^{3+}+H_{2}O_{2}\to {Fe}^{2+}+HO_{2}^{•}+H^{+}$$

Ferrous ion consumption is higher than its production rate. Furthermore, hydroxyl radicals have a rate constant of 3.2–4.3 X 108 M^−1^ s^−1^, which allows them to quickly degrade ferrous ions.^[46]^

(7) $${Fe}^{2+}+HO^{•}\to {Fe}^{3+}+OH^{-}$$

Therefore, in order to maintain the formation of hydroxyl radicals at a safe level, a greater quantity of ferrous ions is necessary. A significant amount of ferric hydroxide sludge is produced during the Fenton process’ neutralization stage, requiring further separation and disposal techniques.

However, in addition to EF technology capacity to efficiently remove pollutants, energy consumption should also be taken into account. To fairly compare EF procedures with other traditional treatment techniques, this is required. The utilization of the PEF method shows promise in eliminating pollutants from industrial wastewater while also accurately measuring power consumption. However, there is a lack of information on the application of this technique. Based on the literature review, the current study compared the H_2_O_2_, electro–Fenton (EF), and pulsed–electro–Fenton (PEF) processes in terms of color and COD removal efficiency as well as consumption of power from industrial wastewater. Operational factors such as COD, pH, H_2_O_2_, distance between electrodes, current, cycle pulse duty, treatment duration, and stirring speed have been investigated to improve the % color and COD removal efficiency with the least amount of power consumption utilizing the PEF method.

## Material and Methods

### Wastewater Collection and Analysis

The wastewater was collected from the distillery industrial sector in Addis Ababa, Ethiopia, using a grab sample method. The wastewater from the distillery was subjected to analysis to determine its quality characteristics, including pH: 4.2–4.4, color:dark brown, BOD: 7000–8000 mg L^−1^, COD: 80,000–90,000 mg L^−1^, odor: burnt sugar, TS: 14.44 g L^−1^, and TDS: 5,450–5,650 mg L^−1^ in accordance with the conventional analysis approach.^[47]^ In the studies, several chemicals were utilized, such as Ag_2_SO_4_, HgSO_4_, HCl, H_2_SO_4_, NaOH, K_2_Cr_2_O_7_, and (NH_4_)_2_Fe(SO_4_)_2_.6H_2_O, etc. All of the chemicals were purchased from Merck, and the analytical reagent (AR) grade was utilized in its original form whenever it was used.

### Experimental Configuration

**Figure 1:**
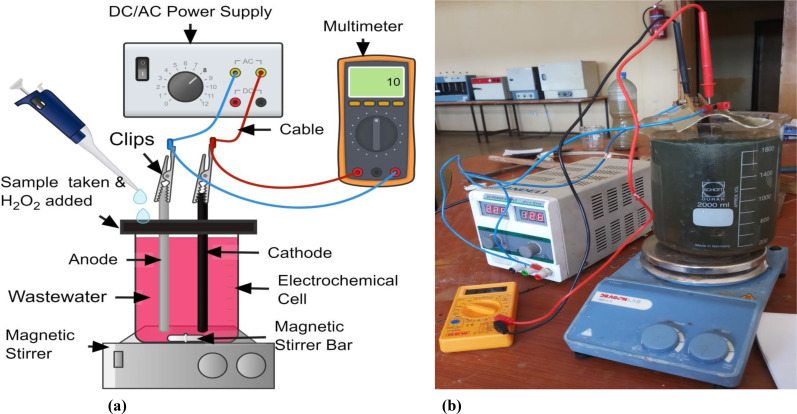
Pulsed‐ Electro‐Fenton (PEF) process experimental setup, (**a)**. Schematic, (**b)**. Actual.

Figure 1 shows the setup of the pulsed–electro–Fenton (PEF) method used to treat wastewater. The batch construction of the EC reactor made of a Perspex glass with a capacity of 2.20 L and wastewater with an active working volume of 2.0 L. The essential COD content of the wastewater was obtained by dilution of raw industrial effluent with distilled water. Before beginning the experiments, the original pH of the wastewater was determined using a pH meter (Elico: Model LI120) and adjusted with 0.1 N H_2_SO_4_ and 0.1 N NaOH solution to the desired level in the range 1–5. As anode and/or cathode, an iron (Fe: grade MS 104) plate with dimensions of 12 cm*15 cm*0.1 cm was employed. In terms of effective electrode surface area, the dimensions were 12 cm by 11.50 cm by 0.1 cm. The bottom of the electrode and the reactor of the EF cell were kept 1 cm apart to provide sufficient stirring. Between 0.75 and 3 cm, the distance between the anode and the cathode was altered in the electrode spacing. The electrodes were cleaned with distilled water and 15 % HCl before to the start of each experiment. A DC/AC power supply (0–5 A, 0–270 V, 50 Hz; AMETEK Model: EC 1000 S) was connected to the anode and cathode in monopolar parallel, and the current was controlled via galvanostatic operation. To determine the power consumption throughout the required treatment duration, the cell voltage was measured using a multimeter. The external introduction of H_2_O_2_ was used to carry out the EF/PEF technique. All studies were conducted at a constant temperature of 35±1 °C. At predefined time intervals, samples were taken from the reactor and centrifuged (REMI, Model: R‐24) for 15 minutes at 15,000 rpm before being tested for color and COD elimination.

### Analysis

#### Elimination Effectiveness, (%)

Before and after the H_2_O_2_, EF, and PEF treatment procedures, the color and COD of industrial wastewater were used to calculate the efficacy of the removal, which was expressed as a percentage. A closed reflux method (Spectroquant® TR 320) and a UV/Vis–Spectrophotometer (Spectroquant® Pharo 300) were utilized in order to determine the COD and color of the sample, adhering closely to the APHA standard method.^[47]^

The efficacy of % color and % COD removal were determined using Equations (8–9.
(8)

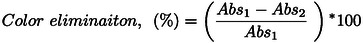




Where, *Abs_1_* and *Abs_2_* are the absorbance of industrial wastewater before and after treatment at a constant wavelength (*λ_max_*).
(9)

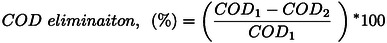




Where, *COD_1_* and *COD_2_* are the COD (mgL^−1^) of industrial wastewater before and after treatment, respectively.

#### Power Consumption

Consumption of power is a significant issue in the EF/PEF process, both practically and economically[^48^, ^49^] and it was computed using the Equation 10.

(10) $$Consumption\;of\;power=\left(\frac{VIt}{V_{R}}\right),\;kWhr/m^{3}$$

Where, V represents voltage (Volts), I denotes current (Amperes), t indicates the duration of EF/PEF treatment (min), and *V_R_* signifies the volume of wastewater employed (liters).

#### Pulse Duty Cycle (θ)

Equation (12) was used to obtain θ, which is defined as the ratio of power on time to total reaction cycle time.^[50]^

(11) θ=(Powerontime/Wholereactioncycletime)

(12)

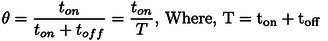




#### Water Recovery, (m^3^/m^3^)

The treatment of wastewater and industrial effluent can involve a variety of methods, including EC, advanced oxidation, biological, chemical, and physical processes,^[51]^ among others, water recovery is an essential characteristic that must be considered. The volume of product water after treatment was compared to the volume of starting wastewater before treatment, and the Equation (13) was used to calculate the ratio between the two.

(13) $$Water\;recovery=\frac{{\left(Volume\;of\;product\;water\right)}_{after\;treatment}}{{\left(Volume\;of\;initial\;wastewater\right)}_{before\;treatment}},\;\left(m^{3}/m^{3}\right)$$

## 2 Results and Discussion

### 2.1 H_2_O_2_, EF and PEF Method Comparison

**Figure 2:**
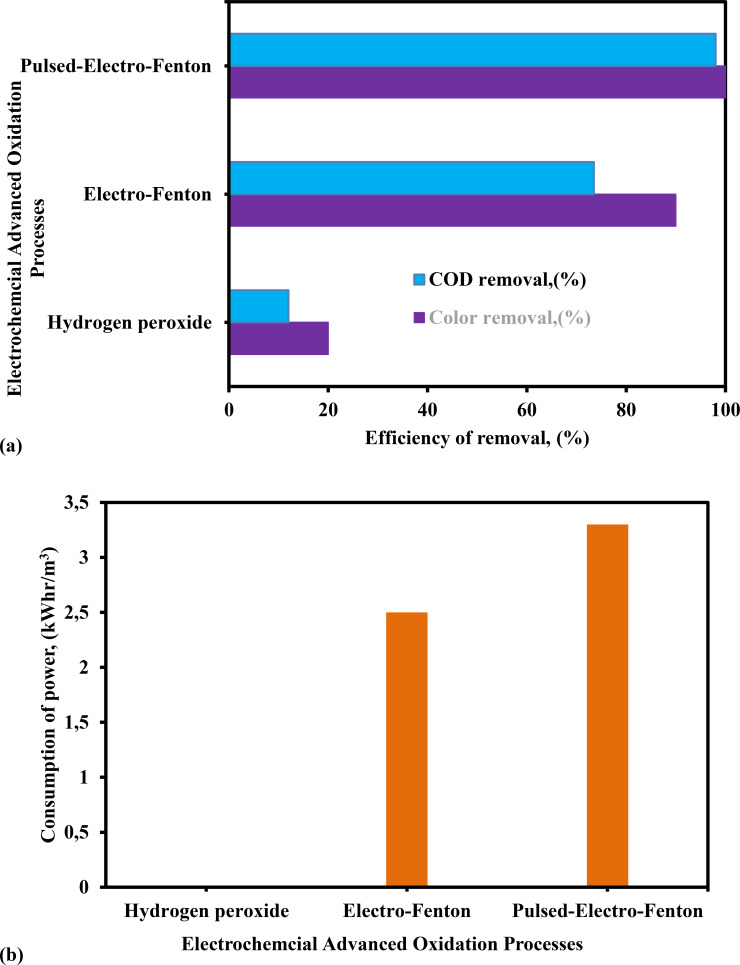
Assessment of H_2_O_2_, EF and PEF methods on (a) efficiency of color and COD elimination, (%) and (b) consumption of power (COD – 2500 mg L^−1^, pH – 3, H_2_O_2_ – 300 mg L^−1^, distance between electrodes (DBE) – 0.75 cm, current −0.40 A, cycle pulse duty (CPD) – 0.75, treatment duration (TD) – 125 min and stirring speed (SS) – 500 rpm).

In the first part of the study, the H_2_O_2_, EF, and PEF processes were evaluated in terms of color and COD elimination efficiency (%), as well as consumption of power from the wastewater. The results are displayed in Figure 2(a). According to Figure 2(a), the PEF method had the most performance in removing both color and COD at a rate of 100 % and 98 %, and the following the decreasing sequence of PEF>EF>H_2_O_2_ process. The results showed that after 125 min of reaction time, the percentage of color and COD elimination efficiency was removed. According to these findings, the PEF technology may simultaneously produce hydroxyl and other chemical oxidants through various procedures. These highly oxidant species promote pollutant removal by (i) speeding anode dissolution by chemical oxidation and (ii) increasing pollutant abatement through the oxidation action of radical species.[^43^, ^46^, ^52^, ^53^]

Within the context of the utilization of EC and AOP technologies for the removal of pollutants from wastewater, the quantity of energy that is used is a component that is of the utmost importance and significance. On the basis of the efficiency of COD elimination, the consumption of power was computed using Equation (10), and the results are shown in Figure 2 (b). As can be seen in Figure 2(b), the consumption of power was around 0, 2.5 and 3.3 kWhr m^−3^ for the H_2_O_2_ alone, EF and PEF, respectively. According to the findings, it is clear that the PEF process required power input of 3.3 kWhr m^−3^ in order to remove 100 % of the color and 98 % of the COD from wastewater. Figure 2(a) and (b) clearly demonstrate that the % of color and COD elimination from wastewater was greater, while consumption of power was lower in the PEF process compared to the EF process. In the case of the PEF process, the amount of sludge that was produced and the formation of the impermeable layer were substantially lower than those of the EF process.[^35^, ^37^, ^49^, ^54^]

The Equation (13) was used to calculate water recovery. Following the EF and PEF processes, while the settled sludge was at the bottom of the electrochemical reactor, the clear supernatant made up the treated water or product water. Table 2 has the display of the findings, when compared to the EF process, the water recovery of the PEF method was significantly more substantial, as indicated by the results of Table 2. Consequently, while evaluating the EF and PEF processes for the removal of percentage color and percentage COD in relation to power consumption from industrial effluent, the PEF approach proved to be more advantageous than the EF process.^[51]^

**Table 2:** Experimental conditions with different initial COD concentration for % color, % COD reduction and consumption of power in EF and PEF processes.

Current (A)	Initial COD concentration (mg L^−1^)	Treatment duration (min)	Final pH	Elimination efficiency, (%)		Consumption of power, (kWhr m^−3^)	Water recovery, (m^3^/m^3^)	
				Color	COD		Directcurrent	Pulsed– Current
0.30	750	125	7.10	95	81.50	2.10	0.85	0.90
0.40	2500	125	7.25	100	98	3.30	0.82	0.91

### 2.2 Studies on Operating Parameters

Process parameters such as COD, pH, H_2_O_2_, distance between electrodes, current, cycle of pulse duty, strring speed and treatment duration on the wastewater PEF process was examined to identify the optimal parameter conditions for maximizing % COD elimination while minimizing power usage. The subsequent section addresses the impact of these operating parameters on the PEF process.

#### 2.2.1 Influence of TD

**Figure 3:**
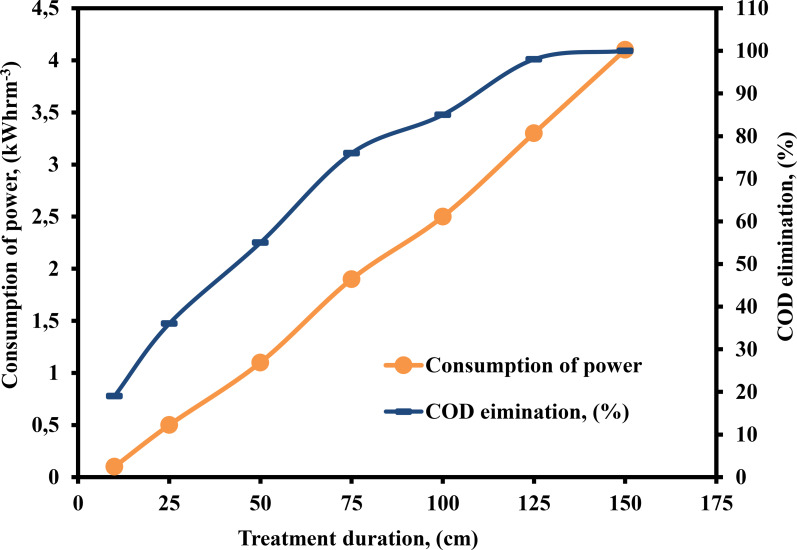
Influence of TD on the consumption of power and COD elimination (%) by PEF method (COD – 2500 mg L^−1^, pH – 3, H_2_O_2_ – 300 mg L^−1^, DBE – 0.75 cm, current – 0.40 A, CPD – 0.75 and SS – 500 rpm).

The treatment duration of the EF method is a significant aspect in determining its economic viability. According to Faraday's Law, the quantity of iron released into the hybrid system through the Fe electrodes can alter the residence duration, resulting in an increase in the number of Fe ions released into the system.[^55^, ^56^] Figure 3 illustrates the impact of TD on the amount of electricity consumed and the efficiency with which COD is eliminated using the PEF technique. The studies were carried out by varying the treatment time from 10–150 min while keeping other parameters constant. Figure 3 indicate that increasing the TD (10‐ 150 min) enhanced the COD elimination efficiency (19–100 %) and increased consumption of power (0.1–4.1 kWhrm^−3^). This was primarily owing to the fact that as treatment time increased, more metal (iron) polymeric species were produced. As a consequence, more ⋅OH were generated, which improved COD removal efficiency.^[57]^ Figure 3 depicts the influence of TD on consumption of power. The consumption of power increased with TD, as seen in Figure 3. TD resulted in an increase in cell voltage, and power consumption was proportional to cell voltage.

#### 2.2.2 Effect of H_2_O_2_

**Figure 4:**
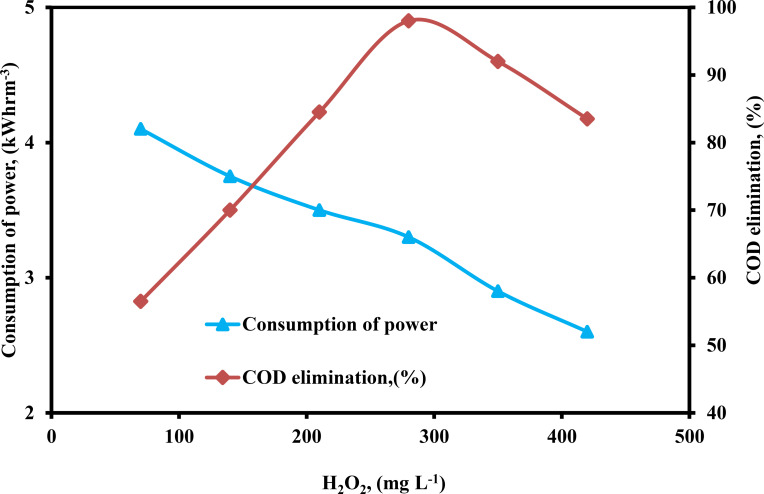
Influence of H_2_O_2_ on the consumption of power and COD elimination (%) by PEF method (COD – 2500 mg L^−1^, pH – 3, DBE – 0.75 cm, current – 0.40 A, CPD – 0.75, TD – 125 min and SS – 500 rpm).

The concentration of H_2_O_2_ is an important factor in the EF process.[^44^, ^46^] The concentration of H_2_O_2_ was changed from 70–420 mg L^−1^ to investigate the influence of H_2_O_2_ concentration on COD elimination efficiency and consumption of power utilizing the PEF process, and the findings are shown in Figure 4. By increasing H_2_O_2_ from 70–280 mg L^−1^, the effectiveness of COD elimination increased from 56.50–98 % while consumption of power decreased from 4.1–3.3 kWhrm^−3^. The reason for this is that there was inadequate generation of ⋅OH, when there was a lower concentration of H_2_O_2_, which resulted in a very slow elimination of COD. Since more ⋅OH was formed as a result of the higher H_2_O_2_ concentration, the quantity of COD that was removed from the water also increased.^[58]^ But further increasing H_2_O_2_ from 280–420 mg L^−1^, the effectiveness of COD removal decreased from 98–83.50 % while consumption of power decreased from 3.3–2.6 kWhrm^−3^. Nevertheless, the % of COD elimination was not discernible at high H_2_O_2_ concentrations. This is because H_2_O_2_ has a scavenging action, inhibits iron corrosion, and recombines hydroxyl radicals. Therefore, 280 mg L^−1^ was determined to be the ideal H_2_O_2_ concentration for removing the most COD while using the least amount of electricity.

#### 2.2.3 Influence of CPD

**Figure 5:**
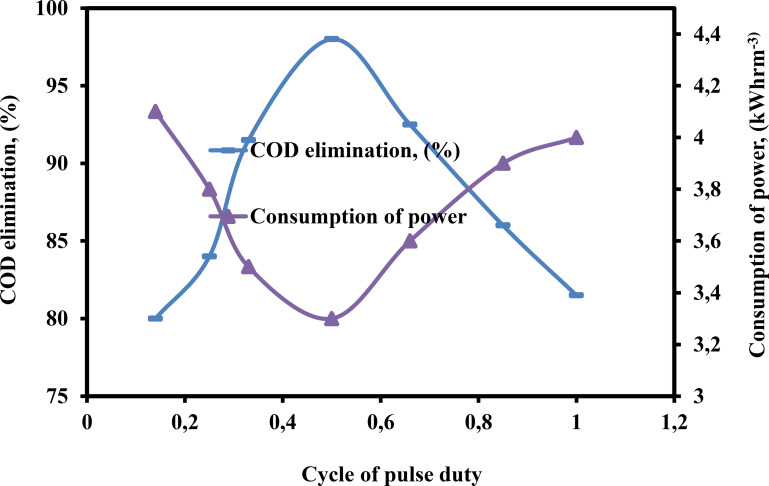
Influence of CPD on the consumption of power and COD elimination (%) by PEF method (COD – 2500 mg L^−1^, pH – 3, H_2_O_2_ – 300 mg L^−1^, DBE – 0.75 cm, current – 0.40 A, TD – 125 min and SS – 500 rpm).

The cycle pulse duty is a key component of the PEF technique for treating wastewater contamination.^[50]^ The results of an evaluation of the impact of CPD on the consumption of power and the % of COD elimination from wastewater using the PEF method are displayed in the Figure 5. The figure makes it clear that consumption of power dropped from 4.1–3.3 kWhr m^−3^ and the % of COD elimination increases from 80–98 % when the CPD increased from 0.14–0.50. As the CPD increases from 0.50–1, consumption of power escalated from 3.3–4 kWhrm^−3^, while the % of COD elimination diminished from 98–81.50 %. The experimental findings indicated that both lower and higher values of the CPD yielded equivalent % of COD elimination and consumption of power in the PEF compared to the EF process. In contrast to other CPD values that were shorter and higher, the generation of coagulant was stronger when the CPD was 0.50. This was the case even when the sludge output and cell voltage were both decreased. Therefore, in order to achieve a high % of COD removal while minimizing the amount of power that is consumed, the CPD should be operated at 0.50 in the PEF process for the treatment of wastewater.

#### 2.2.4 Effect of Current

**Figure 6:**
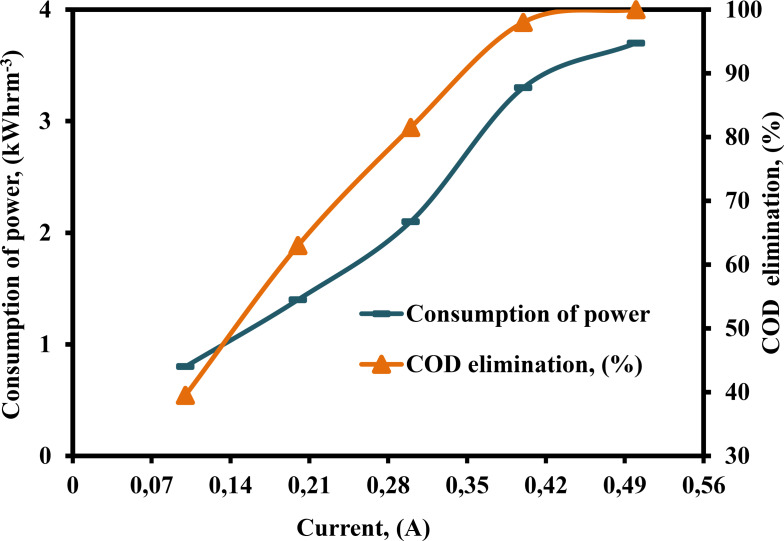
Influence of current on the consumption of power and COD elimination (%) by PEF method (COD – 2500 mg L^−1^, pH – 3, H_2_O_2_ – 300 mg L^−1^, DBE – 0.75 cm, CPD – 0.75, TD – 125 min and SS – 500 rpm).

The quantity of ⋅OH produced and the reaction rate are both affected by current, which is an important part of EF processes.[^44^, ^59^, ^60^] The applied current has a major effect on both the amount of electricity consumed and the efficiency of pollution removal.^[61]^ Current was varied from 0.10–0.50 A to examine on COD elimination efficiency and consumption of power. The data is shown in Figure 6, according to Figure, increased COD elimination efficiency from 39.50–98 % and consumption of power from 0.8–3.3 kWhrm^−3^ with increasing current from 0.1–0.4 A, respectivley. This tendency may be explained by the production of more ⋅OH in the solution as the current increases.^[62]^ The lower current value mandates a longer period for pollutants removal, necessitating larger facilities and higher operational costs. As a result, higher current values lead to energy loss from partial heating caused by electrical energy, as well as higher consumption of power and operating costs. As a result, the current should be optimized to improve pollutant removal effectiveness while using the least amount of power.

#### 2.2.5 Effect of pH

The wastewater pH has a major impact on the EF process, especially in Fenton chemistry.[^63^, ^64^] The pH of the solution significantly impacts the efficacy of EC processes such as electro‐oxidation and EF. The stability of H_2_O_2_, the rate at which OH radicals are generated, and the specific type and form of iron that precipitates out of a solution are all directly impacted by the pH of the solution.^[65]^ Acidic pH values (between 2 and 4) are ideal for the EF method best outcomes.^[64]^ The oxidation potential at this pH range is developed as a result of the accelerated HO⋅ formation, which enables the efficient speciation of iron and the production of H_2_ that are suitable for the Fenton reaction.^[66]^ The process becomes less effective at high pH values, especially above 5, because structural instability causes H_2_O_2_ to break down rapidly into H_2_O and CO_2_ in alkaline environments. Furthermore, the potential for oxidation of ⋅OH is reduced as pH increases.^[65]^

**Figure 7:**
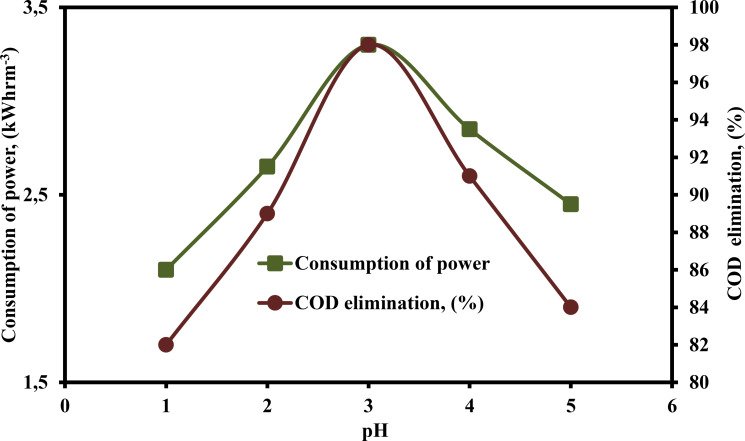
Influence of pH on the consumption of power and COD elimination (%) by PEF method (COD – 2500 mg L^−1^, H_2_O_2_ – 300 mg L^−1^, DBE – 0.75 cm, current – 0.40 A, CPD – 0.75, TD – 125 min and SS – 500 rpm).

The efficacy of COD elimination was analyzed in the range of pH 1–5 (Figure 7). The subsequent parameters were implemented to execute this section of the investigation: COD – 2500 mg L^−1^, H_2_O_2_ – 300 mg L^−1^, DBEs – 0.75 cm, current −0.40 A, CPD – 0.75, TD – 125 min and SS – 500 rpm. The findings indicate that pH has a major influence on both consumption of power and COD elimination efficiency. The pH increased from 1–3, increased the consumption of power and COD elimination efficiency and from 2.1–3.3 kWhm^−3^ and 82–98 %, respectively. Further raising the pH from 3–5 (Figure 7), decreased the consumption of power and COD removal efficiency from 3.3–2.45 kWhrm^−3^ and 98–84 %, respectively. Based on our observations, it appears that the oxidation potential of ⋅OH has a tendency to decrease as the pH increases. When the pH level is high, the development of insoluble ferric hydroxo complexes occurs, which results in the inhibition of the generation of ⋅OH. The Fe^3+^ precipitated in the form of amorphous Fe(OH)_3_ at pH levels greater than 3. The production of Fe(OH)_3_ not only reduced the concentration of dissolved Fe^3+^, but it also slowed Fe^2+^ regeneration by partially covering the electrode surface. For EAOPs, the pH level of 3 was shown to be the optimal value for the most efficient elimination of pollutants.

#### 2.2.6 Effect of COD

**Figure 8:**
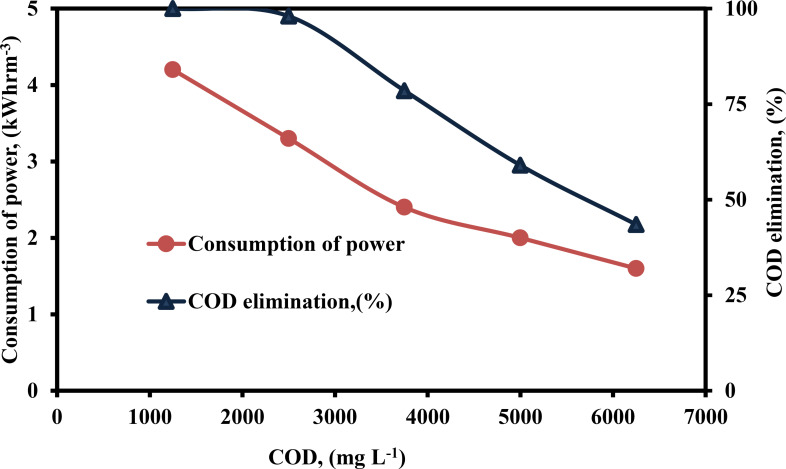
Influence of COD on the consumption of power and COD elimination (%) by PEF method (pH – 3, H_2_O_2_ – 300 mg L^−1^, DBE – 0.75 cm, current – 0.40 A, CPD – 0.75, TD – 125 min and SS – 500 rpm).

Figure 8 show the results of changing the wastewater of CODs from 1250–6250 mg L^−1^ to explore the influence of COD concentration on consumption of power and COD elimination efficiency using the PEF process. The consumption of power (4.2–1.6 kWhrm^−3^) and efficiency of COD removal (100–43.50 %) was decreased as COD concentration increased (1250–6250 mg L^−1^). One of the characteristics of EF processes is this behavior, Faraday's Law states that for any value of constant current and treatment period, an increasing initial COD concentration results in the release of a constant amount of Fe^2+^ into the solution. As COD concentration increased in an electrolysis experiment, specific quantities of ⋅OH were produced. Notwithstanding, the quantity of ⋅OH produced was insufficient to decompose the high concentration of COD in wastewater.[^67^, ^68^] In summary, when the COD content rose, the oxidizing effectiveness decreased.

#### 2.2.7 Effect of DBE

**Figure 9:**
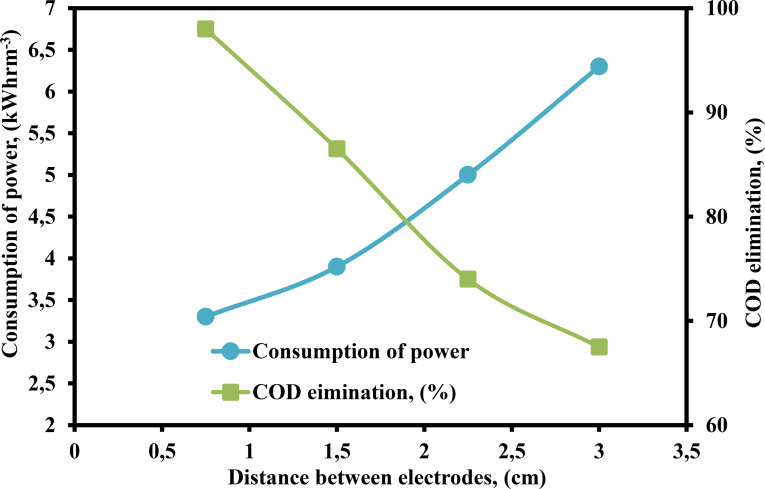
Influence of DBE on the consumption of power and COD elimination (%) by PEF method (COD – 2500 mg L^−1^, pH – 3, H_2_O_2_ – 300 mg L^−1^, current – 0.40 A, CPD – 0.75, TD – 125 min and SS – 500 rpm).

An experiments were carried out in which the distance between the anode and the cathode was changed from 0.75–3 cm by utilizing the PEF technique. Figure 9 provides a schematic representation of the findings. By increasing the distance between the anode and the cathode, the consumption of power increased from 3.30–6.30 kWhrm^−3^, and the % of COD removal reduced from 98–67.50 %. As a result of the increased distance between the anode and the cathode, the electrical current was reduced, the voltage was raised, and the IR drop was multiplied. Additionally, the reduced interaction of ions with hydroxide polymer and electrostatic attraction are factors that impact the percentage of COD removal as well as the consumption power of distillery effluent.^[29]^ The inter‐electrode spacing between the anode and cathode was optimized to 0.75 cm in order to obtain a higher percentage of COD elimination with minimal power consumption from the PEF process.

#### 2.2.8 Effect of SS

**Figure 10:**
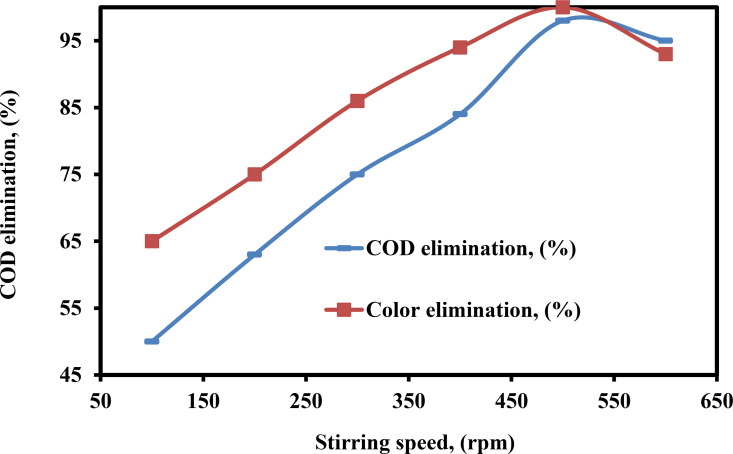
Influence of SS on the consumption of power and COD elimination (%) by PEF method (COD – 2500 mg L^−1^, pH – 3, H_2_O_2_ – 300 mg L^−1^, DBE – 0.75 cm, current – 0.40 A, CPD – 0.75 and TD – 125 min).

The Figure 10 illustrates how wastewater color and COD removal are affected by rotating stirring speed. The % of color (65–100 %) and COD (50–98 %) elimination increases with rotational speed up to 500 rpm, then decreases as rpm increases from 500–600 rpm. This is because there are two regimes: one sets the speed between 0 and 500 rpm, and the other sets the speed higher. Through the use of gentle stirring, the mixing efficiency between the color bodies and the hydrolyzed Fe^3+^ is improved, which ultimately results in an increase in the percentage of color elimination.^[69]^ Additionally, gentle stirring may evenly disperse H_2_ bubbles throughout the solution, increasing the flocs’ capacity to float.^[70]^ The coagulated color molecules in the solution are dispersed when the rotational speed is increased to more than 500 rpm, which leads to a reduction in the % of color and COD that is attached to iron hydroxide.

### 2.3 UV/ViS‐ Spectrophotometer Analysis for Color Removal

**Figure 11:**
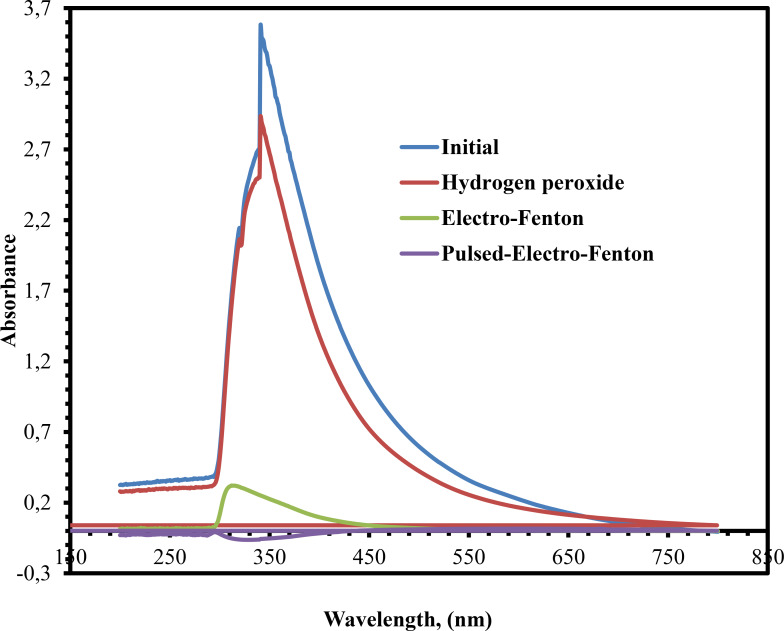
UV/ViS‐Spectrophotometer analysis.

The UV/Vis‐spectrophotometer used for the evaluation of effluent from the distillery industry is shown in Figure 11 for the purpose of color determination. After the application of the H_2_O_2_, EF and PEF process, it was recorded in comparison to the wastewater that was produced by the distillery at the beginning of the process. Figure 11 demonstrated that the absorbance spectrum of the distillery industrial effluent had an absorbance peak at 300 nm, which was attributed to the coloring agent. The absorbance of peaks experienced a substantial decrease following the treatment processes, as illustrated in Figure 11. It might be a result of the formation of intermediates that occurs when color and COD are removed. In conlusion, the results of the UV/Vis‐Spectra experiments, which can be seen in figure demonstrated that the PEF process was more effective than the EF and H_2_O_2_ method when it came to the removal of color from industrial effluent from distilleries.

## 3 Conclusions

According to the findings of this study, the H_2_O_2_, EF, and PEF technologies were used to treat actual industrial wastewater from distilleries. The % of color and COD eliminated, as well as the consumption of power were compared for each of these processes. In comparison to other treatment approaches, the PEF process eliminated 100 % of the color and 98 % of the COD with consumption of power of 3.30 kWhr m^−3^. In the optimal experimental conditions, the PEF process exhibited a lower consumption of power and a higher % of color and COD elimination compared to the EF process. The % COD elimination and consumption of power from wastewater by means of an PEF technique were influenced by COD, pH, H_2_O_2_, distance between electrodes, current, cycle of pulse duty, strring speed and treatment duration. When compared to the EF process, the PEF method resulted in a much smaller amount of sludge generation and exceptionally high water recovery. The results of the experiments have demonstrated that the PEF process is an innovative method that is highly effective in the removal of contaminants. Additionally, it is suitable to all types of wastewater as well as effluent from industrial processes.

## 4 Funding

This research was funded by the Researchers Supporting Project number (RSP2025R54), King Saud University, Riyadh, Saudi Arabia.

## Author Contributions

Perumal Asaithambi: Investigation; Data curation; Resources; Writing – original draft.

Abdelrahman O. Ezzat: Conceptualization; Methodology; Validation; Supervision.

Firomsa Bidira: Investigation; Data curation; Formal analysis; Resources.

Mamuye Busier Yussuf: Investigation; Data curation; Formal analysis; Resources.

Omar H. Abd‐Elkader: Conceptualization; Methodology; Validation; Supervision.

Arun Thirumurugan: Conceptualization; Methodology; Validation; Supervision.

N. M. Hariharan: Conceptualization; Methodology; Validation; Supervision.

Hamad A. Al‐Lohedan: Conceptualization; Methodology; Validation; Supervision.

Abudukeremu Kadier: Conceptualization; Methodology; Validation; Supervision.

## Conflict of Interests

The authors declare that they have no known competing financial interests or personal relationships that could have appeared to influence the work reported in this paper.

## Data Availability

The authors do not have permission to share data.

## References

[1] B. M. Omar , M. A. Zyadah , M. Y. Ali , M. A. El-Sonbati , Sci. Rep. 2024, 14, 27906. 39537851 10.1038/s41598-024-78846-w PMC11561168

[2] A. Mahdi , M. Arshadi , M. Baghdadi , G. Nabi bidhendi , J. Environ. Manage. 2024, 372, 123221. 39571312 10.1016/j.jenvman.2024.123221

[3] M. Mohammadi , R. Davarnejad , M. Sillanpää , Results Eng. 2024, 24, 102938.

[4] A. Y. Ghjeer , A. H. Abbar , Case Stud. Chem. Environ. Eng. 2023, 8, 100519.

[5] N. S. da Cruz Santana Neves , A. L. A. de Lucena , V. de Oliveira Marques Cavalcanti , B. R. Galdino , J. M. Rodríguez-Díaz , M. M. M. B. Duarte , M. Benachour , D. C. Napoleão , Catal. Commun. 2024, 186, 106828.

[6] N. Oturan , J. Bo , C. Trellu , M. A. Oturan , ChemElectroChem 2021, 8, 3294–3303.

[7] J. Ghodsi , A. A. Rafati , R. A. Joghani , ChemistrySelect 2021, 6, 8889–8898.

[8] M. Al-Shannag , Z. Al-Qodah , K. Alananbeh , N. Bouqellah , E. Assirey , K. Bani-Melhem , Environ. Eng. Manage. J. 2014, 13, 3153–3160.

[9] W. Tang , N. Ma , C. Fei , Y. Wang , ChemistrySelect 2022, 7, e202200313.

[10] N. M. Mahmoodi , A. Dalvand , Desalin. Water Treat. 2013, 51, 5959–5964.

[11] K. Thirugnanasambandham , V. Sivakumar , J. Prakash Maran , Environ. Prog. Sustain. Energy 2015, 34, 411–419.

[12] M. Mirzaei , K. Moazeni , M. Baghdadi , A. Aliasghar , N. Mehrdadi , Int. J. Environ. Sci. Technol. 2024, 21, 8391–8401.

[13] D. Rai , S. Sinha , Chemosphere 2023, 336, 139225. 37356583 10.1016/j.chemosphere.2023.139225

[14] J.-Z. Wang , T.-H. Ha , C. Chiemchaisri , M.-C. Lu , J. Water Proc. Eng. 2024, 63, 105504.

[15] A. A. Moneer , W. M. Thabet , M. Khedawy , M. M. El-Sadaawy , N. A. Shaaban , Int. J. Environ. Sci. Technol. 2023, 20, 13859–13872.

[16] A. G. Merma , B. F. Santos , A. S. C. Rego , R. R. Hacha , M. L. Torem , J. Mater. Res. Technol. 2020, 9, 15164–15176.

[17] F. Ilhan , K. Ulucan-Altuntas , Y. Avsar , U. Kurt , A. Saral , Front. Environ. Sci. Eng. 2019, 13, 73.

[18] B. Chezeau , L. Boudriche , C. Vial , A. Boudjemaa , Sep. Sci. Technol. 2020, 55, 2510–2527.

[19] A. A. Mohamud , Y. Çalışkan , N. Bektaş , H. C. Yatmaz , Sep. Sci. Technol. 2018, 53, 2468–2475.

[20] S. Najari , M. Delnavaz , D. Bahrami , Chem. Phys. Lett. 2023, 832, 140897.

[21] K. E. Adou , A. R. Kouakou , A. D. Ehouman , R. D. Tyagi , P. Drogui , K. Adouby , Sci. Afr. 2022, 16, e01238.

[22] F. Özyonar , M. U. Korkmaz , Chemosphere 2022, 290, 133172. 34914950 10.1016/j.chemosphere.2021.133172

[23] M. A. A. Hamid , H. A. Aziz , M. S. Yusoff , Sustain. Solut. Environ. Pollut. 2021, 1, 257–304.

[24] Z. Qi , S. You , R. Liu , C. J. Chuah , Front. Environ. Sci. Eng. 2020, 14, 40.

[25] A. Aygun , B. Nas , M. F. Sevimli , Korean J. Chem. Eng. 2019, 36, 1441–1449.

[26] F. Ozyonar , B. Karagozoglu , Int. J. Environ. Sci. Technol. 2012, 9, 637–646.

[27] R. K. Patel , R. Shankar , P. Khare , P. Mondal , J. Indian Chem. Soc. 2022, 99, 100563.

[28] Y. G. Asfaha , F. Zewge , T. Yohannes , S. Kebede , Chemosphere 2022, 302, 134706. 35523291 10.1016/j.chemosphere.2022.134706

[29] N. Modirshahla , M. A. Behnajady , S. Kooshaiian , Dyes Pigm. 2007, 74, 249–257.

[30] F. Janpoor , A. Torabian , V. Khatibikamal , J. Chem. Technol. Biotechnol. 2011, 86, 1113–1120.

[31] B. yul Tak , B. sik Tak , Y. ju Kim , Y. jin Park , Y. hun Yoon , G. ho Min , J. Ind. Eng. Chem. 2015, 28, 307–315.

[32] L. E. Quispe Cardenas , P. J. Deptula , C. S. Huerta , C. Zhu , Y. Ye , S. Wang , Y. Yang , ACS EST Eng. 2023, 3, 1547–1556. 10.1021/acsestengg.3c00128 PMC10580281 37854076

[33] A. Varindani , T. S. Anantha Singh , P. Menon , P. V. Nidheesh , Environ. Technol. (U.K.) 2022, 43, 3497–3506. 10.1080/09593330.2021.1923819 33944690

[34] M. Eyvaz , Int. J. Electrochem. Sci. 2016, 11, 4988–5008.

[35] A. Othmani , A. Kesraoui , M. Seffen , Euro-Mediterranean J. Environ. Integr. 2017, 2, DOI 10.1007/s41207-017-0016-y.

[36] P. Asaithambi , R. Govindarajan , M. B. Yesuf , P. Selvakumar , E. Alemayehu , J. Environ. Chem. Eng. 2021, 9, 104811.

[37] S. Vasudevan , J. Lakshmi , G. Sozhan , J. Hazard. Mater. 2011, 192, 26–34. 21612863 10.1016/j.jhazmat.2011.04.081

[38] L. Xu , Q. Huang , X. Xu , G. Cao , C. He , Y. Wang , M. Yang , Sep. Purif. Technol. 2017, 188, 316–328.

[39] H. J. Mansoorian , A. H. Mahvi , A. J. Jafari , Sep. Purif. Technol. 2014, 135, 165–175.

[40] T. Xu , Y. Zhou , B. Hu , X. Lei , G. Yu , Ecotoxicol. Environ. Saf. 2020, 197, 110629. 32325329 10.1016/j.ecoenv.2020.110629

[41] F. Rajaei , E. Taheri , S. Hadi , A. Fatehizadeh , M. M. Amin , N. Rafei , S. Fadaei , T. M. Aminabhavi , Environ. Pollut. 2021, 277, 116632. 33640826 10.1016/j.envpol.2021.116632

[42] E. Gatsios , J. N. Hahladakis , E. Gidarakos , J. Environ. Manage. 2015, 154, 117–127. 25721979 10.1016/j.jenvman.2015.02.018

[43] H. A. Alalwan , N. S. Mohammed Ali , M. M. Mohammed , M. F. Mohammed , A. H. Alminshid , Clean. Eng. Technol. 2023, 13, 100623.

[44] A. R. Yazdanbakhsh , M. R. Massoudinegad , S. Eliasi , A. S. Mohammadi , J. Water Proc. Eng. 2015, 6, 51–57.

[45] İ. A. Şengil , S. Kulaç , M. Özacar , J. Hazard. Mater. 2009, 167, 940–946. 19237242 10.1016/j.jhazmat.2009.01.099

[46] S. Farhadi , B. Aminzadeh , A. Torabian , V. Khatibikamal , M. Alizadeh Fard , J. Hazard. Mater. 2012, 219–220, 35–42. 10.1016/j.jhazmat.2012.03.013 22464981

[47] APHA, Standard Methods for the Examination of Water and Wastewater, 1496, **2012**.

[48] A. Dalvand , M. Gholami , A. Joneidi , N. M. Mahmoodi , Clean Soil Air Water 2011, 39, 665–672.

[49] M. Alimohammadi , A. Mesdaghinia , M. H. Shayesteh , H. J. Mansoorian , N. Khanjani , Int. J. Environ. Sci. Technol. 2019, 16, 8239–8254.

[50] M. Ren , Y. Song , S. Xiao , P. Zeng , J. Peng , Chem. Eng. J. 2011, 169, 84–90.

[51] E. Keshmirizadeh , S. Yousefi , M. K. Rofouei , J. Hazard. Mater. 2011, 190, 119–124. 21531074 10.1016/j.jhazmat.2011.03.010

[52] V. K. Sandhwar , B. Prasad , Korean J. Chem. Eng. 2018, 35, 909–921.

[53] W. Zhang , M. Zhang , J. Yao , J. Long , Arab. J. Chem. 2023, 16, 104607.

[54] Y. Bian , Z. Ge , C. Albano , F. L. Lobo , Z. J. Ren , Environ. Sci. Water Res. Technol. 2019, 5, 1654–1660.

[55] P. Maha Lakshmi , P. Sivashanmugam , Sep. Purif. Technol. 2013, 116, 378–384.

[56] P. Asaithambi , M. B. Yesuf , R. Govindarajan , N. M. Hariharan , P. Thangavelu , E. Alemayehu , J. Environ. Manage. 2022, 320, 115926. 35940007 10.1016/j.jenvman.2022.115926

[57] G. Divyapriya , R. Srinivasan , J. Mohanalakshmi , I. M. Nambi , J. Water Proc. Eng. 2022, 49, 102967.

[58] K. Thirugnanasambandham , S. Kandasamy , V. Sivakumar , R. K. Kumar , R. Mohanavelu , J. Taiwan Inst. Chem. Eng. 2015, 46, 89–97.

[59] N. Masomboon , C. Ratanatamskul , M.-C. Lu , Appl. Catal. A 2010, 384, 128–135.

[60] S. Garcia-Segura , A. El-Ghenymy , F. Centellas , R. M. Rodríguez , C. Arias , J. A. Garrido , P. L. Cabot , E. Brillas , J. Electroanal. Chem. 2012, 681, 36–43.

[61] E. Pajootan , M. Arami , M. Rahimdokht , Ind. Eng. Chem. Res. 2014, 53, 16261–16269.

[62] M. S. Çelebi , N. Oturan , H. Zazou , M. Hamdani , M. A. Oturan , Sep. Purif. Technol. 2015, 156, 996–1002.

[63] P. Menon , T. S. Anantha Singh , N. Pani , P. V. Nidheesh , Chemosphere 2021, 269, 128739. 33131740 10.1016/j.chemosphere.2020.128739

[64] S. Şahinkaya , J. Ind. Eng. Chem. 2013, 19, 601–605.

[65] A. Dargahi , M. Moradi , R. Marafat , M. Vosoughi , S. A. Mokhtari , K. Hasani , S. M. Asl , Biomass Convers. Biorefin. 2021, DOI 10.1007/s13399-021-01753-x.

[66] S. Vasudevan , J. Water Proc. Eng. 2014, 2, 53–57.

[67] M. Sedaghat , B. Vahid , S. Aber , M. H. Rasoulifard , A. Khataee , N. Daneshvar , Res. Chem. Intermed. 2016, 42, 855–868.

[68] J. Wu , W. Pu , C. Yang , M. Zhang , J. Zhang , J. Environ. Sci. 2013, 25, 801–807. 10.1016/s1001-0742(12)60117-x 23923790

[69] M. Susanna James, A. Garg, *Chemosphere* **2024**, *346*, DOI 10.1016/j.chemosphere.2023.140572. 38303390

[70] I. Khatri , S. Singh , A. Garg , J. Environ. Chem. Eng. 2018, 6, 7368–7376.

[71] L. Nouri Sarabi , S. Shariati , A. Islamnezhad , H. Kefayati , Water Air Soil Pollut. 2024, 235, 546.

[72] S. F. Alturki , M. S. Suwaed , A. Ghareeb , F. Y. AlJaberi , A. A. Hassan , J. Eng. Res. 2024, DOI 10.1016/j.jer.2024.10.006.

[73] A. Mohmmad , M. T. Hamed Mosavian , M. H. Haddad Khodaparast , Int. J. Environ. Sci. Technol. 2024, 21, 35–42.

[74] I. Ahmad , D. Basu , Iran. J. Sci. Technol. Trans. Civ. Eng. 2024, 48, 1715–1729.

[75] A. Shokri , B. Nasernejad , Process Saf. Environ. Prot. 2023, 172, 836–845.

[76] M. M. Jiad , A. H. Abbar , Case Stud. Chem. Environ. Eng. 2023, 8, DOI 10.1016/j.cscee.2023.100431.

[77] A. Y. Ghjair , A. H. Abbar , Process Saf. Environ. Prot. 2023, 169, 481–492.

[78] A. Kuleyin , A. Gök , F. Akbal , J. Environ. Chem. Eng. 2021, 9, DOI 10.1016/j.jece.2020.104782.

